# Whispering Gallery Mode Enabled Efficiency Enhancement: Defect and Size Controlled CdSe Quantum Dot Sensitized Whisperonic Solar Cells

**DOI:** 10.1038/s41598-018-27969-y

**Published:** 2018-06-26

**Authors:** Tapan Kumar Das, P. Ilaiyaraja, C. Sudakar

**Affiliations:** Multifunctional Materials Laboratory, Department of Physics, Indian Institute of Technology Madras, Chennai, 600036 India

## Abstract

A synergetic approach of employing smooth mesoporous TiO_2_ microsphere (SμS-TiO_2_)–nanoparticulate TiO_2_ (np-TiO_2_) composite photoanode, and size and defect controlled CdSe quantum dots (QD) to achieve high efficiency (η) in a modified Grätzel solar cell, *quantum dot sensitized whisperonic solar cells (QDSWSC)*, is reported. SμS-TiO_2_ exhibits whispering gallery modes (WGM) and assists in enhancing the light scattering. SμS-TiO_2_ and np-TiO_2_ provide conductive path for efficient photocurrent charge transport and sensitizer loading. The sensitizer strongly couples with the WGM and significantly enhances the photon absorption to electron conversion. The efficiency of QDSWSC is shown to strongly depend on the size and defect characteristics of CdSe QD. Detailed structural, optical, microstructural and Raman spectral studies on CdSe QD suggest that surface defects are prominent for size ~2.5 nm, while the QD with size > 4.5 nm are well crystalline with lower surface defects. QDSWSC devices exhibit an increase in η from ≈0.46% to η ≈ 2.74% with increasing CdSe QD size. The reported efficiency (2.74%) is the highest compared to other CdSe based QDSSC made using TiO_2_ photoanode and I^−^/I_3_^−^ liquid electrolyte. The concept of using whispering gallery for enhanced scattering is very promising for sensitized whisperonic solar cells.

## Introduction

Quantum dots (QD) as a photo-sensitizer for solar cell has gained large attention in the recent years^1–4^. Size dependent bandgap tunability^5,6^, high absorption coefficient (10^5^ cm^−1^)^7^, multiple exciton generation (MEG)^8^, and hot electron injection^9^ are few of the several factors that make QD as a suitable sensitizer for harvesting solar energy in quantum dot sensitized solar cells (QDSSC). Various QD crystals, with most notable ones including CdSe^10,11^, CdS^12,13^, ZnSe^12,14^ and CuInS_2_^15,16^ have been tested as a photo-sensitizer. The broad range of solar energy could be harvested using these semiconductor QD by tuning its bandgap through controlling the size and composition^17,18^. CdSe exhibit excellent optical properties with the absorption edge tailorable over the whole range of visible spectrum and therefore has been studied extensively in QDSSC devices^19–21^. The absorption coefficients of CdSe QD dispersed in chloroform and hexane are reported to be around 1.48 × 10^5^ cm^−1^ and 1.33 × 10^5^ cm^−1^ respectively, which is very high compared to other QD^22^. The efficiency of QDSSC also depends on the quality of photoanode, effective loading of sensitizer on photoanode and charge transport across the junction. In spite of various advantages, solar cell devices made using QD have shown significantly lower efficiency than the theoretically expected values due to various reasons. QD synthesis procedures invariably use size controlling organic surfactants^23^. The presence of long chain surfactant on QD surface limits the effective loading of QD on TiO_2_ and also suppresses the effective charge transfer across the junction of TiO_2_/QD interface in solar cell^24–27^. Advances in QD surface passivation to reduce the defect by ligand exchange mechanism have led to rapid improvements in QDSSC power conversion efficiency^3^. To maximize QD loading on photoanode and activate effective charge transport across the junction of TiO_2_/QD interface, the long chain surfactants on the QD surface are replaced by conducting linker molecules. Molecules such as 3-mercaptopropionic acid (MPA) and atomic ligand such as S^2─^ are used to link the QD with TiO_2_ photoanode^3^.

For sensitized solar cells (SSC), photonanode is also equally important for deciding the performance of solar cell^28^. The anatase phase of TiO_2_ has been used as photoanode in SSC^29^. After Grätzel’s seminal work on dye-sensitized solar cells using mesoporous TiO_2_ photoanode, the research on improving the microstructure of TiO_2_ got escalated^30^. Suitable morphology of TiO_2_ with good optical properties such as high light scattering is preferable for photoanode. In our recent work, we established that the composite photoanode structure comprising of mesoporous microspherical TiO_2_ along with nanoparticle in an optimized ratio (80:20) provide higher light absorption and charge transport leading to enhanced efficiency in dye sensitized solar cell^31^. Such composite anode exhibiting whispering gallery modes (WGM), used in a modified Grätzel type solar cells called ‘whisperonic solar cells’ were shown to enhance the efficiency of sensitized solar cells. Further, the charge transfer between QD and TiO_2_ is driven mainly by the difference in the band alignment between them^2^. The band alignment of QD depends on its size as the position of conduction band of QD with respect to conduction band of TiO_2_ vary with QD size^11^. Thus the QD size is one of the most important factors that determine the charge transfer rate. The size of QD should be optimized for maximum light absorption and electron injection to enable maximum solar energy conversion efficiency. Short-band gap semiconductor can harvest solar spectrum effectively if assembled on photoanode with a proper alignment of energy levels. Earlier studies have investigated the size dependent solar energy conversion efficiency of CdSe QD based QDSSC^32^. However, a maximum PCE of only 0.8% is reported for MPA capped CdSe QD using I^−^/I_3_^−^ liquid electrolyte in QDSSC devices^32^. Low PCE is attributed to the poor absorption of solar light and fast charge recombination thus limiting electron harvesting. Hence, significant effort is being pursued to optimize the size dependent properties of CdSe QD and photoanode structure.

In this manuscript, we report a detailed study on the optical, microstructural and structural properties of CdSe QD synthesized in the size range between 2.5 nm to 5.2 nm using a surfactant controlled growth process. Surface defects are shown to be more prominent in the smaller CdSe QD of size ~2.5 nm, while QD with size >4.5 nm are shown to be well crystalline with lower surface defects. Quantum dot sensitized whisperonic solar cells (QDSWSC) were fabricated by loading CdSe QD of different sizes on a composite photoanode made of mesoporous smooth microsphere (SμS-TiO_2_) and nanoparticulate-TiO_2_^33^. Whispering gallery modes (WGM), a prominent Mie scattering phenomena present in SμS-TiO_2_ lead to large light scattering and further strongly couple with the CdSe QD sensitizer. A maximum efficiency of 2.74% for CdSe based QDSWSC is reported for QD of size ~4.7 nm. This measured efficiency is higher than reported earlier for CdSe QD sensitized solar cells using I^−^/I_3_^−^ liquid electrolyte.

## Results and Discussion

**Figure 1:**
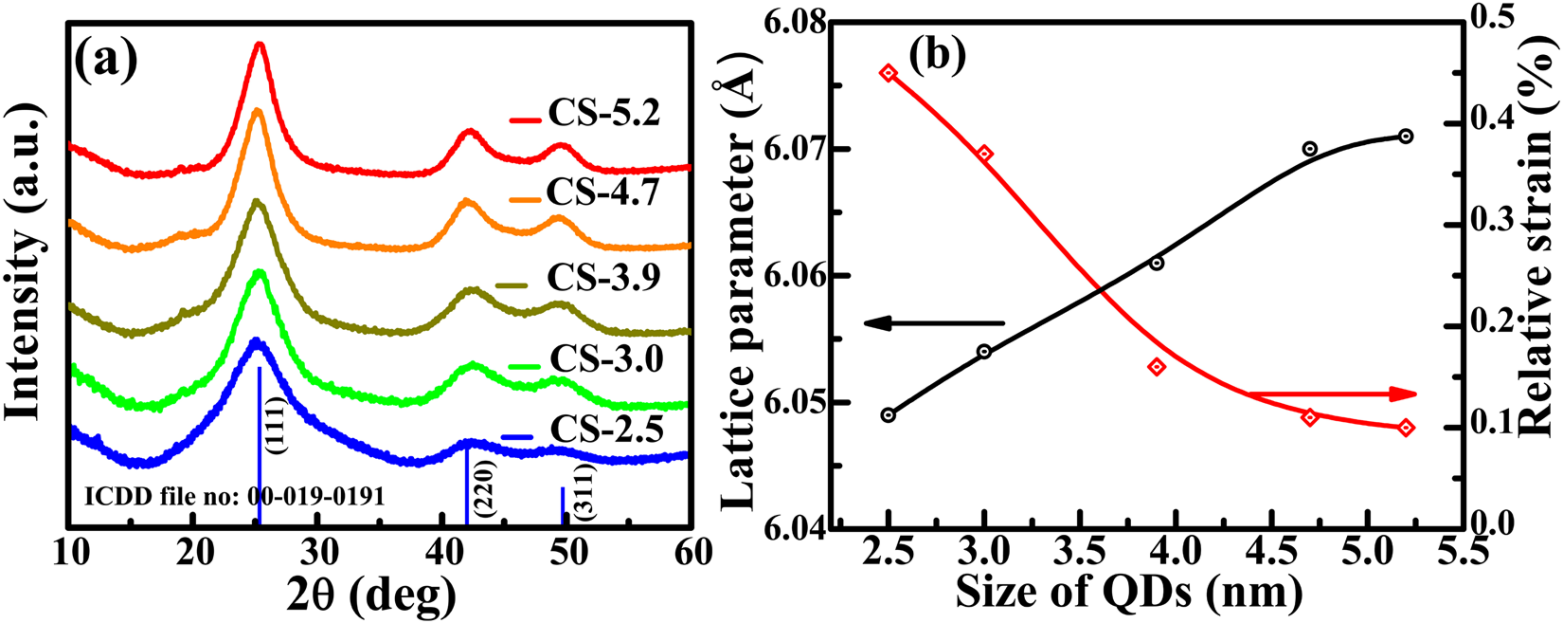
(**a**) X-ray diffraction patterns of CdSe quantum dots of various sizes. The vertical lines are the standard ICDD file (00-19-0191) based CdSe peak positions shown with relative peak intensities. (**b**) Estimated lattice parameter and relative strain $({\rm{\Delta }}a/a)$) in % due to lattice contraction as a function of QD size.

**Figure 2:**
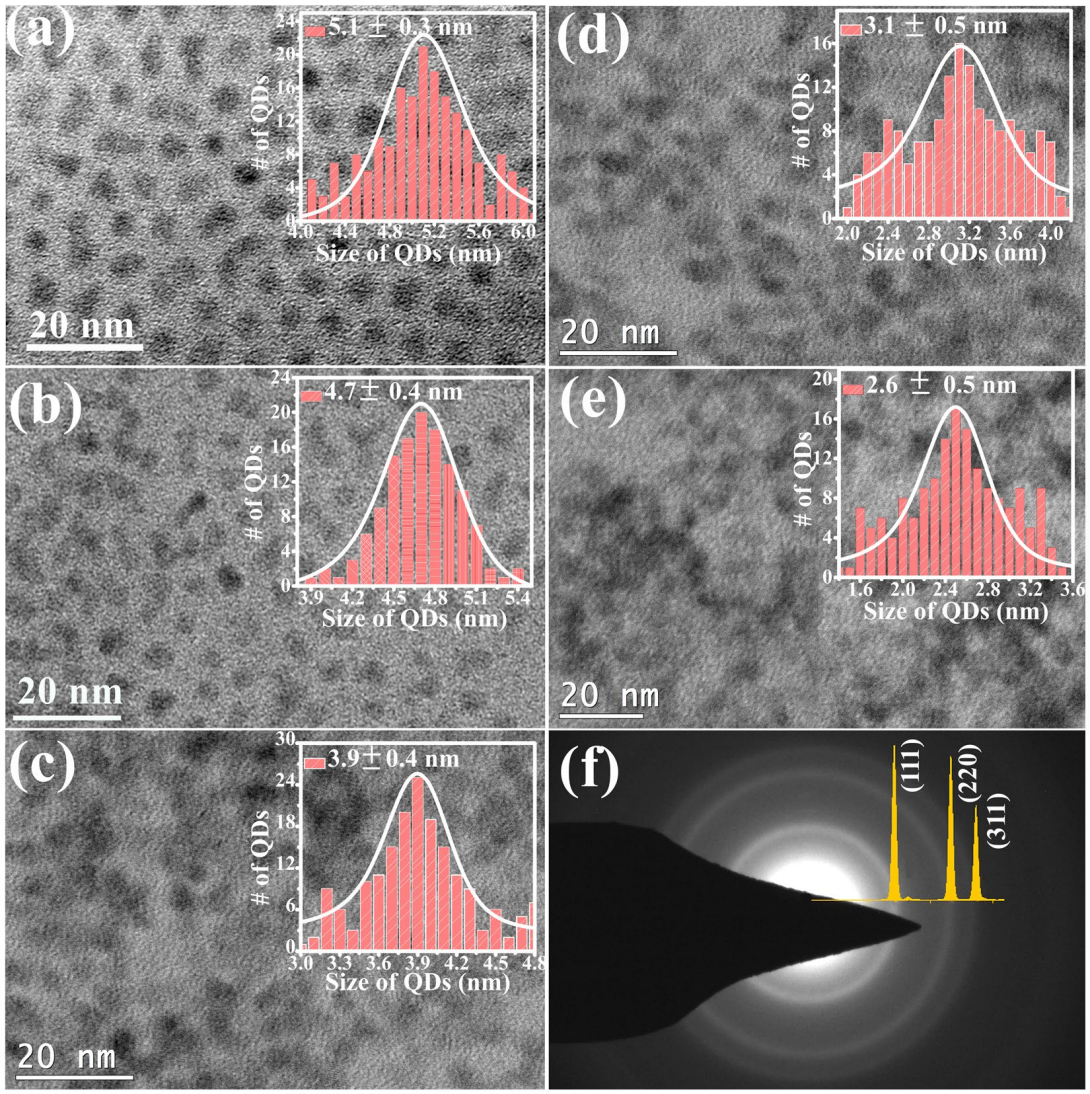
Bright field TEM image of CdSe QD: (**a**) CS-5.2, (**b**) CS-4.7, (**c**) CS-3.9, (**d**) CS-3.0, and (**e**) CS-2.5. The size distribution histogram plots are shown in the inset of the corresponding panel in (**a**) to (**e**). The average size with the standard deviation from the Gaussian fit is indicated in the insets. The SAD pattern of a representative sample of CS-5.2 and the simulated peak profile of CdSe zincblende structure is shown in (**f**).

Crystallographic phase of CdSe QD for various sizes as inferred from X-ray diffraction (XRD) are shown in Fig. 1a. All the XRD peaks are indexed to a cubic zincblende structure in accordance with the ICDD file no: 00-019-0191. CdSe QD form phase pure zincblende structure. The broad X-ray reflection of (111), (220) and (311) planes clearly suggest the sizes of QDs are in sub-nanometer range. The full-width-at-half maximum (FWHM) of these diffraction peaks become narrow with increasing size of QD. The crystalline size of QD estimated using Debye-Scherrer equation is found to be in the range of ~2.5 to ~5.2 nm. The XRD peak positions of QD are also found to be slightly off from the bulk CdSe, with the peak shifting towards lower 2θ angles with increase in QD size, indicating a contraction of lattice in smaller QD. The lattice parameters of CdSe QD are estimated from the peak position of (111), (220) and (311) reflections. The change in the estimated lattice parameter ‘*a*’ and the relative strain $({\rm{\Delta }}a/a)$ as a function of size is shown in Fig. 1b. The lattice parameter of CS-2.5 sample is *a* = 6.049 Å, much lower than the bulk value (*a* = 6.077 Å). As the QD size increases to 5.2 nm, lattice parameter increases to *a* = 6.071 Å approaching values close to the bulk (see Table 1). This confirms the relaxation of compressive strain present in the smaller QD. The lattice contraction is found to be ~0.10% for CS-5.2 QD, however for smaller QD e.g. CS-2.5, ${\rm{\Delta }}a/a$ has much higher values of 0.44% (see Fig. 1b). Since the QD were grown in a solution medium without any substrate, the lattice contraction could mainly arise due to the surface reconstruction. The lattice contraction reported by Hwang *et al*.^34^ was found when the CdSe QD are embedded in borosilicate glass substrate. The lattice contraction in such cases arise from the mismatch of thermal expansion coefficient between nanocrystal and the host medium, in addition to the contributions from surface tension which increases with decreasing nanocrystal size^35,36^. Zhang *et al*.^37^ also observed the lattice contraction in free-standing CdSe nanocrystals of wurtzite structure. This was attributed to the surface optimization/reconstruction during the growth^37^. Further investigation of structural, microstructural and size distribution were carried out using TEM studies of CdSe QD (Fig. 2). The size distribution of QD calculated from the TEM images exhibit Gaussian distribution and the average size is found to be consistent with the crystallite size estimated from XRD studies. These QD exhibit a uniform distribution in size and shape. The size of QD, lattice parameters and lattice strain calculated from XRD are given in Table 1.

**Table 1:** CdSe quantum dots of various sizes prepared by hot-injection method.

Sample code	Crystallite size (nm) XRD	Particle size (nm) TEM	Lattice parameter (Å)	Lattice strain (%)
CS-2.5	2.5 ± 0.2	2.6 ± 0.5	6.049(8)	0.44 ± 0.11
CS-3.0	3.0 ± 0.2	3.1 ± 0.5	6.054(6)	0.38 ± 0.10
CS-3.9	3.9 ± 0.2	3.9 ± 0.4	6.061(5)	0.26 ± 0.08
CS-4.7	4.7 ± 0.2	4.7 ± 0.4	6.070(4)	0.11 ± 0.04
CS-5.2	5.2 ± 0.2	5.1 ± 0.3	6.071(4)	0.10 ± 0.03

The size estimated from the XRD, TEM along with the crystallite size, lattice parameter and lattice strain estimated from XRD are given. The error in the estimation of crystallite size from XRD, and the estimated standard deviation in the particle size from TEM studies and lattice parameter from XRD are given in the parenthesis.

**Figure 3:**
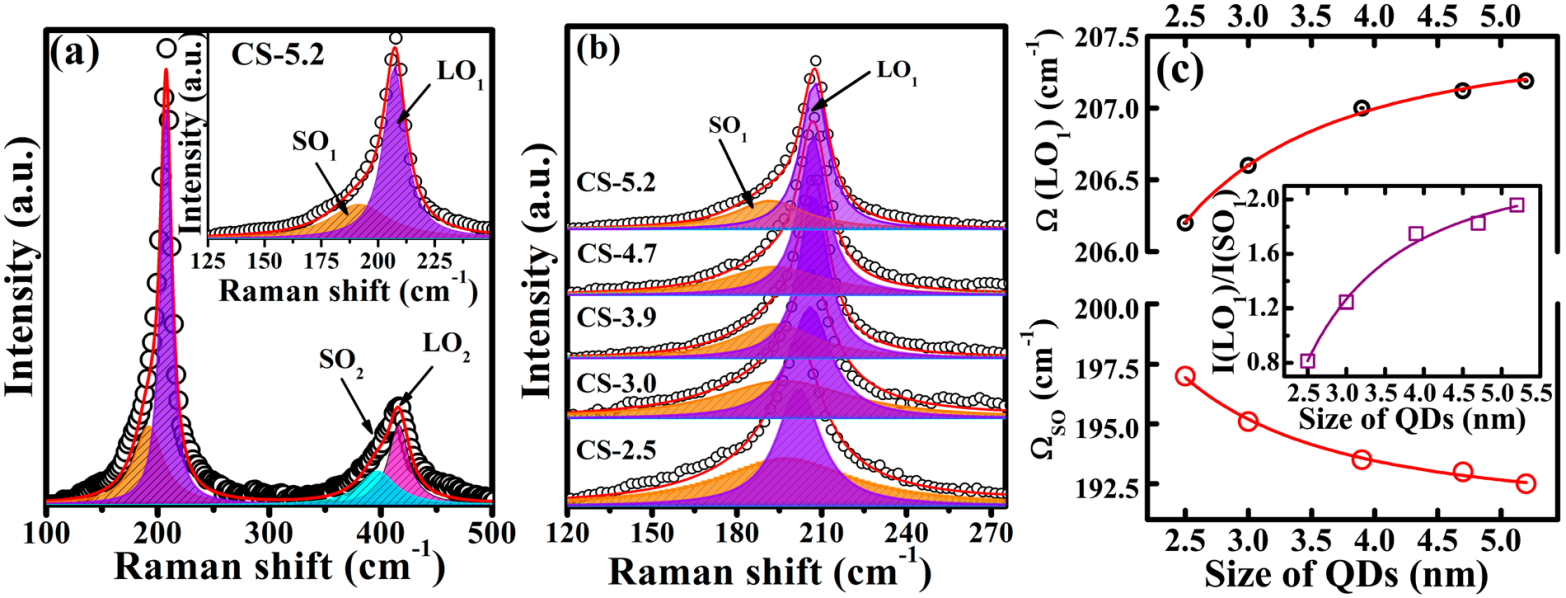
(**a**) Raman spectrum of CdSe QD of size 5.2 nm. The spectrum is fitted (red line) with Lorentzian profiles shown in the shaded colors. The first-order optical phonons showing the LO_1_ and SO_1_ phonon modes are clearly shown in the magnified plot given in the inset. (**b**) LO_1_ and SO_1_ phonon modes for various sizes of CdSe quantum dots. The spectra show the fitted curves (red line) using two Lorentizian function for surface and longitudinal optical phonon modes. (**c**) Raman shift of LO_1_ and SO_1_ phonon modes as a function of QD size with corresponding fits using the equations (2) and (5) given in the text. Inset (**c**) show the ratio of integrated intensity of LO_1_ and SO_1_ phonon modes in the Raman spectra of CdSe QD. The line is a guide to eye. All the spectra were recorded at 300 K using an excitation source of 488 nm.

The size dependent local structural changes of CdSe QD are studied by Raman spectra using 488 nm laser line excitation from Ar-ion laser (output power ~ 1 mW). Since the crystal symmetry of CdSe is C_*6v*_, only A_1_, E_1_ and E_2_ symmetry modes are Raman active. The most dominant asymmetric Raman mode seen with peak position between ~204 cm^−1^ to ~208 cm^−1^ is from the first order longitudinal optical phonon (LO_1_) (Fig. 3a). Its overtone LO_2_ phonon modes is seen at ~408 cm^−1^. Both LO phonon modes are fitted with Lorentizian peak profile. A representative fit for the Raman spectrum of 5.2 nm CdSe QD is shown in Fig. 3a. Due to the asymmetric broadening in LO_1_ mode, the spectra could be completely fitted only if the profile corresponding to a surface optical phonon (SO) mode is included^36^. A magnified part of the spectrum showing the fits for the SO_1_ and LO_1_ optical phonons is shown in the inset of Fig. 3a. Size dependent Raman spectra of CdSe quantum dots are shown in Fig. 3b. Good fits for the observed asymmetric phonon modes of various sizes of QD are obtained by taking the sum of two Lorentizian functions, one for LO and the other for SO modes. In all the Raman spectra LO_1_ and LO_2_ phonon modes of the CdSe are clearly seen at ~200 cm^−1^ and at ~420 cm^−1^, respectively. The position of LO_1_ phonon modes for CS-2.5 QD is at 206.2 cm^−1^ which is red shifted by 3.8 cm^−1^ with respect to bulk value of 210 cm^−1^. A systematic red shift from ~ 3.8 cm^−1^ to 2.8 cm^−1^ in LO_1_ phonon mode is observed with increase in the CdSe QD size from 2.5 nm to 5.2 nm (Fig. 3c and Table 2). Further the peak positions are found to shift according to the confinement model proposed by Campbell *et al*.^38^. The LO_1_ phonon frequency shifts to the lower frequency and the peak becomes broader with decreasing QD size (Figs 3c and S1 in Supporting Information). The phonon frequency of the QD are predicted by the phonon dispersion based on the relaxation of the q = 0 selection rule or the Fröhlich polar interactions^39,40^. Thus, the shift in LO phonon mode could originate from two sources: (i) a red shift in LO phonon due to confinement of the optical phonons $({\rm{\Delta }}{\omega }_{D}(R))$ and (ii) a blue shift caused by lattice contraction $({\rm{\Delta }}{\omega }_{C}(R))$. The net shift in LO frequency of QDs as a function of size *R* is give as^41^;

1 $$\omega (R)={\omega }_{L}+{\rm{\Delta }}{\omega }_{D}(R)+{\rm{\Delta }}{\omega }_{C}(R)$$

Here ${\rm{\Delta }}{\omega }_{D}(R)$, and ${\rm{\Delta }}{\omega }_{C}(R)$ are the peak shift due to phonon dispersion and lattice contraction respectively, ${\omega }_{L}$ and $\omega (R)$ are the LO phonon frequency of the bulk and the phonon frequency of quantum dots of radius *R*. The overall effect on the LO phonon shift with crystal size is due to (i) strain arising in QD crystals from the crystal surface reconstruction induced by surface tension and (ii) the compressive strain due to phonon dispersion. The size dependent phonon frequency is generally rewritten as given below for the fitting of the experimental data after considering both effect^36^;

2 $${\omega }_{i}(R)={A}_{i}\mp \frac{{B}_{i}}{{R}^{2}}$$

where

$${A}_{i}={\omega }_{i}^{bulk}[1-3{\gamma }_{i}({\alpha }^{i}-\alpha )(T-{T}_{g})]$$

and

3 $${B}_{i}={\omega }_{i}^{bulk}[\frac{1}{2}{(\frac{{\beta }_{i}{\mu }_{i}}{{\omega }_{i}^{bulk}})}^{2}\mp {\gamma }_{i}{k}_{i}b]$$

**Table 2:** The LO and SO peak positions and FWHM obtained from the Raman spectra of CdSe quantum dots of various sizes are given.

Sample code	LO_1_ (cm^−1^)	FWHM (cm^−1^)	SO_1_ (cm^−1^)	FWHM (cm^−1^)	Ratio of I_LO1_/I_SO1_	${\boldsymbol{\Delta }}{{\boldsymbol{\omega }}}_{{\boldsymbol{L}}}{\boldsymbol{(}}{\boldsymbol{R}}{\boldsymbol{)}}$ (cm^−1^)	${\boldsymbol{\Delta }}{{\boldsymbol{\omega }}}_{{\boldsymbol{C}}}{\boldsymbol{(}}{\boldsymbol{R}}{\boldsymbol{)}}$ (cm^−1^)	${\boldsymbol{\Delta }}{{\boldsymbol{\omega }}}_{{\boldsymbol{D}}}{\boldsymbol{(}}{\boldsymbol{R}}{\boldsymbol{)}}$ (cm^−1^)
CS-2.5	206.2	19.4	197	58.2	0.81	3.8	3.0	6.8
CS-3.0	206.6	16	195.1	52.1	1.24	3.4	2.6	6.0
CS-3.9	207	13	193.5	45.1	1.75	3.0	1.1	4.1
CS-4.7	207.1	12.2	193	43.5	1.82	2.9	0.8	3.7
CS-5.2	207.2	11.6	192.5	41.2	1.96	2.8	0.7	3.5

The ratio of integrated intensities of LO_1_/SO_1_ and net shift in the peak ${\rm{\Delta }}{\omega }_{L}$ along with the calculated peak shift due to phonon dispersion ${\rm{\Delta }}{\omega }_{D}(R)$ and lattice contraction ${\rm{\Delta }}{\omega }_{C}(R)$ are given.

$i=LO\,or\,TO$ and $\mp$ sign in equation (2) indicates for LO (−) and TO (+) modes respectively. The ${B}_{i}$ describes the phonon dispersion in bulk crystal, ${\gamma }_{i}$ is the Gruneisen parameter and ${k}_{i}$ is the compressibility for the longitudinal (transverse) mode. α^*i*^ and α are the linear thermal expansion coefficients of the host matrix and QD respectively. The size dependent surface tension of the QD is given as ‘*b*’. *T* and *T*_*g*_ are the measurement and heat treatment temperatures. As cadmium has very large capture cross section for neutrons, very few experimental data on phonon dispersion for CdSe obtained using neutron-scattering techniques are reported. In contrast, from Corso *et al.’s*^42^ calculations using density functional theory, the phonon dispersion curves for various II-VI semiconductor including CdSe with zinc-blende phase show the value of LO_1_ is ~222 cm^−1^, in larger disagreement with the experimental results reported for QD. Due to these differences, Hwang *et al*.^35^ followed the fitting of the experimental data using equation (2) to find the value of ${A}_{i}$ and ${B}_{i}$. For the LO phonon mode, the fitted values of ${A}_{LO}$ and ${B}_{LO}$ are reported as 207.7 cm^−1^ and 11.1 cm^−1^nm^2^ respectively. The values estimated from our data (Fig. 3c) for ${A}_{LO}$ ~ 209.5 cm^−1^ and ${B}_{LO}$ ~ 8.87 cm^−1^nm^2^ are in good agreement with that obtained for CdSe QD in a glass matrix^35^. The fitted plots for LO phonon modes given in Fig. 3c shows good fit obtained by the least-square method. The overall effect on the LO phonon shift with crystal size is due to (i) strain arising in QD crystals surface reconstruction induced by surface tension and (ii) compressive strain resulting from the phonon dispersion.

A quantitative prediction of the magnitude of these two effects can be inferred as discussed below. In order to estimate the lattice contraction induced blue shift $[{\rm{\Delta }}{\omega }_{C}(R)$] in LO frequency, the ${\rm{\Delta }}a/a$ details obtained from XRD is fed in the following equation^35,39,43^,

4 $${\rm{\Delta }}{\omega }_{C}(R)={\omega }_{L}[{(1+3\frac{{\rm{\Delta }}a}{a}(R))}^{-\gamma }-1]\approx -\,3\gamma {\omega }_{L}\frac{{\rm{\Delta }}a}{a}(R)$$

where γ is the Gruneisen parameter and ${\rm{\Delta }}a/a$ is the lattice contraction which also depends on size and temperature. By substituting the value of Gruneisen parameter γ = 1.1 for CdSe, ${\omega }_{L}$= 210 cm^−1^ at 300 K temperature^35,43^, and the ${\rm{\Delta }}a/a$ = −0.0010 estimated from XRD for 5.2 nm size QD, we find that the ${\rm{\Delta }}{\omega }_{C}(R)$ is ~0.7 cm^−1^. Such finding implies that ${\rm{\Delta }}{\omega }_{D}(R)$ contribution to the shift is −3.5 cm^−1^ with overall red shift −2.8 cm^−1^ (Table 2). In the case 2.5 nm QD of size, ${\rm{\Delta }}{\omega }_{C}(R)$ is found to be ~3.0 cm^−1^. This imply a ${\rm{\Delta }}{\omega }_{D}(R)$ contribution to be ~6.8 cm^−1^ with a overall shift of 3.8 cm^−1^. The observed blue shift in LO phonon frequency with the increase of QD size is due to the simultaneous reduction of ${\rm{\Delta }}{\omega }_{D}(R)$ and ${\rm{\Delta }}{\omega }_{C}(R)$, however with the size dependent ${\rm{\Delta }}{\omega }_{D}(R)$ found to be more pronounced than the size dependent ${\rm{\Delta }}{\omega }_{C}(R)$. While both the effects are found to reduce with increasing QD size, they may not completely disappear due to surface effects.

For solar cell device application, it is essential to have good crystalline CdSe QD of optimum size with less surface defects. CdSe quantum dots are prone to get large concentration of surface defects as the size decreases which make SO phonon modes to get activated in the Raman spectra^36^. The surface optical (SO) phonon modes therefore can be used as a gauge to infer the surface defects present in CdSe QD. By analyzing the behavior of SO modes in comparison with LO mode, we decipher the quality of QD and the defect concentrations qualitatively. The SO phonon modes dependence on the shape, size and density of QD can be addressed by two different approaches. The first approach considers the nanostructure as an isolated object and its interaction with the electromagnetic wave is developed by taking into account the dielectric function of the bulk material and the boundary conditions of the electric field at the interface^44^. The second approach considers nanostructure ensembles as a homogeneous material and the interaction between nanostructures and the electromagnetic wave is addressed by effective dielectric function called Fröhlich polar interactions^39,40^. These interactions depend on the TO phonon frequency, and it also changes due to positive phonon dispersion and the lattice contraction. For spherical QD, using the size dependent TO phonon frequency ${\omega }_{TO}(R)$, the size dependent ${\omega }_{SO}(R)$ can be presented by the following equation^36,45,46^,

5 $${\omega }_{SO}(R)={\omega }_{TO}(R){[\frac{{{\epsilon }}_{0}l+{{\epsilon }}_{m}(l+1)}{{{\epsilon }}_{\infty }l+{{\epsilon }}_{m}(l+1)}]}^{1/2}={A}_{SO}+\frac{{B}_{SO}}{{R}^{2}}$$

where ${{\epsilon }}_{0}$ and ${{\epsilon }}_{\infty }$ are the static and high frequency dielectric constants of the bulk CdSe and ${{\epsilon }}_{m}$ is the static dielectric constant of the surrounding medium. ${A}_{SO}$ and ${B}_{SO}$ are the two parameters that depend on the phonon frequency and surface tension^36^. SO phonon mode dependence on R follows the equation (5) for the given angular momentum quantum number *l* = 1 with ${A}_{SO}$ = 190.28 cm^−1^ and ${B}_{SO}$ = 49.1 cm^−1^nm^2^. These values are in good agreement with the values obtained, i.e. ${A}_{SM}$ =190.4 cm^−1^ and ${B}_{SM}$ = 31.9 cm^−1^nm^2^, for CdSe QD embedded in glass matrix^36^. By assuming ${{\epsilon }}_{m}$ = 1 for the bare QDs and using ${\omega }_{TO}(R)$ = 172.0 cm^−1^ for bulk CdSe, the value of ${\omega }_{SO}(R)$ is estimated to be 200 cm^−1^, which is in good agreement with the values reported in the literature^39,46^. The surface optic phonon mode frequency and the ratio of the integrated area of LO_1_ to SO_1_ as a function of QD size are summarized in Fig. 3c. The frequency of SO phonon shifts to the lower frequency with size, in contrast to the LO phonon mode which increases with QD size. Further the SO phonon peak broadens with decreasing QD size (Fig. S1b) and becomes more prominent in smaller QD (Fig. 3c, inset). CdSe QD also show relative change of phonon dispersion and lattice contraction with size. The lattice contraction effect is found to influence SO phonon mode more in the smaller sized QD. This can also be inferred from the ratio of integrated area of LO_1_ to SO_1_ modes, which increases with QD size suggesting that the smaller QD are having more surface defect leading to enhanced SO signal (inset Fig. 3c). This enhancement in SO phonon along with more lattice contraction in smaller size QD confirm the presence of surface disorder deciphered from the XRD and Raman spectral studies.

**Figure 4:**
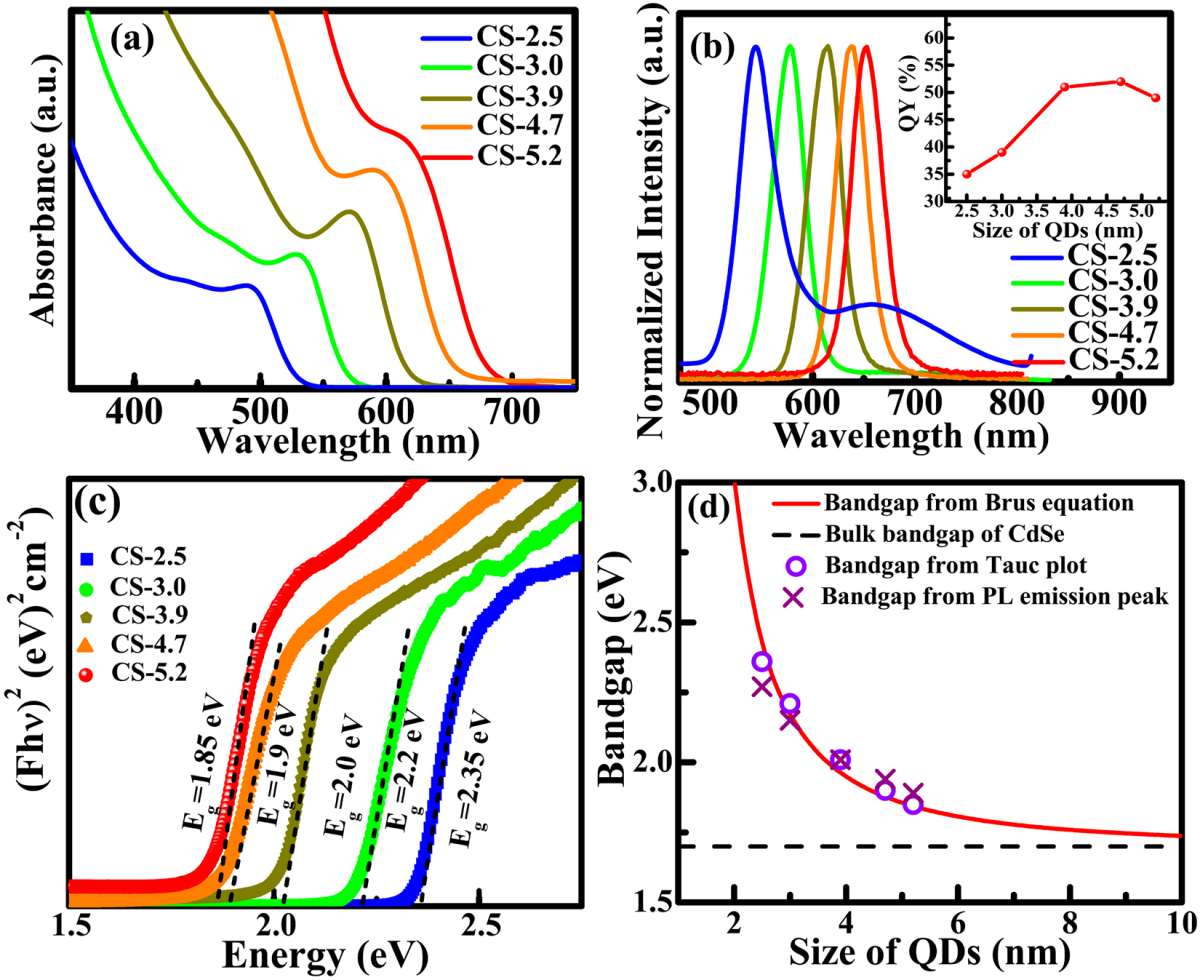
(**a**) Absorption spectra, (**b**) normalized emission spectra, (**c**) Tauc plot obtained from diffuse reflectance spectra and (**d**) the comparison of the experimentally determined bandgap of CdSe QDs with theoretically calculated values using Brus equation. The emission spectra were recorded using an excitation wavelength of 400 nm. The inset in (**b**) shows the value of corresponding quantum yield of different sized QD and the flat line (at 1.7 eV) in (**d**) indicate the bulk bandgap value of CdSe. The bandgap are calculated experimentally from the Tauc plot and the energy from the PL emission peak position (**d**).

The optical absorption and photoluminescence (PL) spectra of CdSe QD of different sizes are shown in Fig. 4a,b. Absorption spectra collected between 350 nm to 750 nm show sharp excitonic peaks indicating the uniform size distribution of QD (Fig. 4a). The excitonic peak position blue shifts with the decrease in QD size pointing out the existence of strong quantum confinement effect in CdSe QD. The sizes of QD can be calculated from absorption spectra using numerical fitting function given by Peng *et al*.^47^. The sizes estimated from this empirical equation are consistent with the average crystallite size derived from XRD and HRTEM studies. The change in the band-edge position of CdSe QD as a function of size can also be inferred from the PL spectra. The PL spectra of QD measured with an excitation wavelength (*λ*_*exc*_) of 400 nm are shown in Fig. 4b. The near-band-edge emission (NBE) of CdSe QD lies between ~520 nm to ~620 nm. The difference in the energy corresponding to the absorption edge and the PL peak, i.e. Stoke shift, is ~20 to 40 nm. Otherwise, the red shift with increasing QD size is similar to that of 1^st^ excitonic peak shift observed in the absorption spectra. Thus, from both the absorption and PL spectra, the quantum confinement effect in CdSe QD is clearly observed. The PL emission peaks are narrow (FWHM ~ 40 nm), thus confirming the formation of uniformly distributed QD. Specifically, for lower sized CdSe QD of size ~2.5 nm, in addition to the NBE emission, a broad peak due to deep defect level (DL) appears. Such a spectral feature reveals the contribution of defect sites to the overall emission. The DL emissions mainly arise from the recombination through surface defect states. However, this DL emission gets suppressed as QD size increases. PL lifetimes of CdSe QD samples at their NBE positions estimated from decay studies using an exponential decay function are in the range of 1.8 ns to 3.5 ns (Fig. S2a). We also estimated the quantum yield (QY) of CdSe QD as a function of size (inset Fig. 4b). The QY increases with particle size initially up to a size of ~4.7 nm and then shows a saturating trend. The maximum value QY is found to be 52% for CdSe QD of average size ~ 4.7 nm. The lower QY for smaller sized QD mainly result from the surface defect states located in the bandgap of the nanocrystals, which could act as trapping states for photo generated charges^48–50^.

The bandgap of CdSe QD are estimated by converting the measured diffuse reflectance spectra into absorption equivalent Kubelka-Munk function. Figure 4c show the Tauc plots derived from diffuse reflectance spectra of CdSe QD in the size range of 2.5 nm to 5.2 nm. The estimated bandgap values of CdSe QD are found to be 2.35 eV, 2.2 eV, 2.0 eV, 1.9 eV and 1.85 eV for the QD sizes of 2.5 nm, 3.0 nm, 3.9 nm, 4.7 nm and 5.2 nm, respectively (Fig. 4c). A plot comparing the experimentally determined bandgap of CdSe QD with theoretically calculated bandgap using Brus equation is shown in Fig. 4d. The bandgap values calculated from the Tauc’s plots match well with the theoretical curve obeying Brus equation^51^. As the size of QD increases the experimentally determined bandgap of CdSe QD become closer to the bulk value of CdSe.

**Figure 5:**
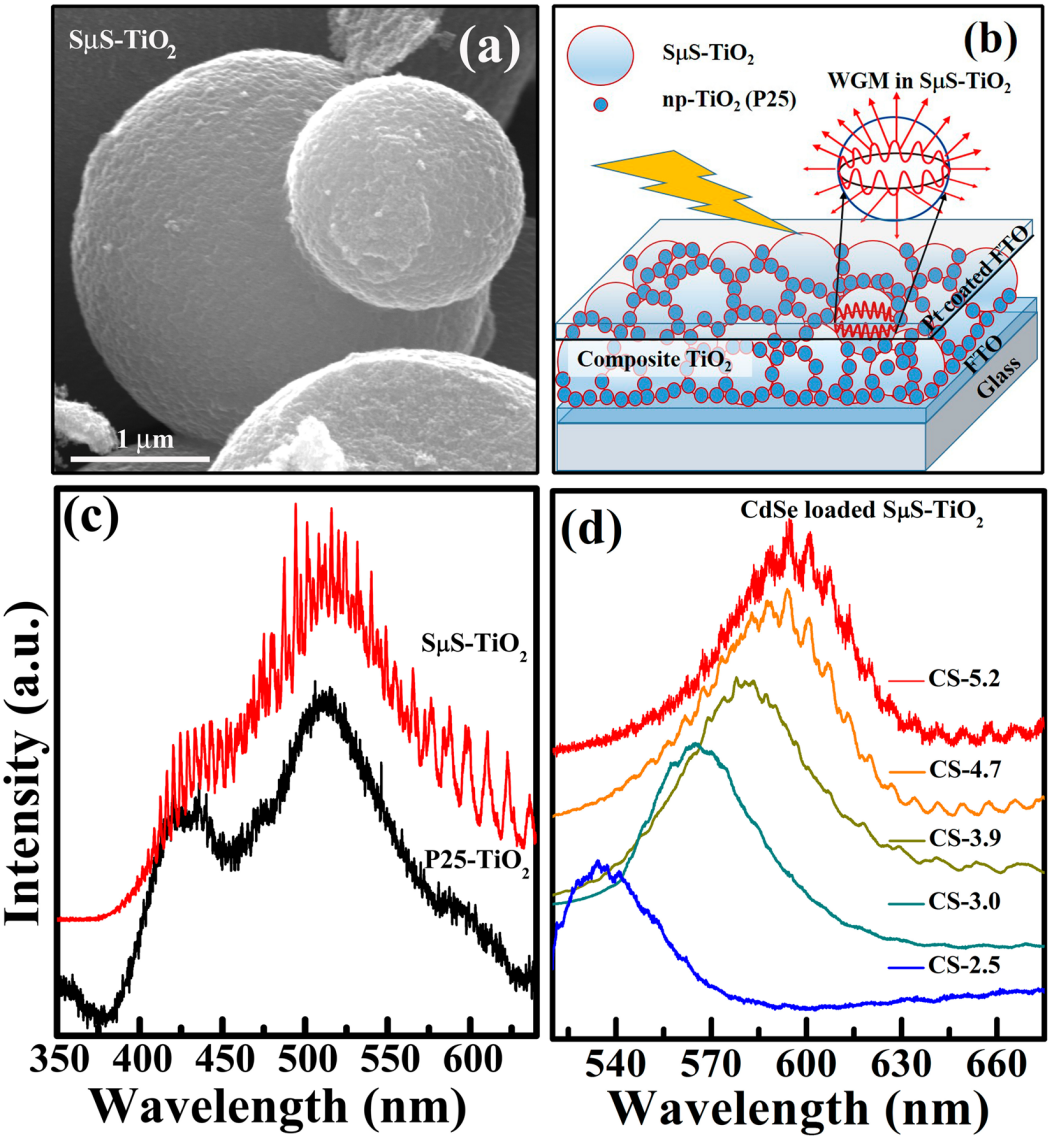
(**a**) FESEM image of SμS-TiO_2_. (**b**) Schematic representation of quantum dots sensitized whisperonic solar cells, also depicting the formation of whispering gallery modes (WGM) in TiO_2_ microsphere (SμS -TiO_2_). (**c**) PL excitation spectra obtained from P25-TiO_2_ and SμS-TiO_2_ using an UV laser excitation source of wavelength λ_exc_ = 325 nm. (**d**) PL excitation spectra of CdSe QD of various sizes loaded on SμS-TiO_2_ obtained using λ_exc_ = 488 nm laser source.

The composite TiO_2_ photoanode, on which the CdSe QD are loaded to fabricate the solar cell, is made using 80 wt.% of SμS-TiO_2_ and 20 wt.% of P25-TiO_2_ nanocrystallites^31^. The morphological and microstructural details of SμS-TiO_2_, P25-TiO_2_ and their composites are analyzed using FESEM images (Figs 5a and S3). This composite photoanode is shown to be very promising for a modified Grätzel type solar cell called whisperonic solar cell^31,33^. The unique advantages of the morphology and the optimized composite ratio useful for DSSC performance were discussed in detail in our previous report^31^. Due to a strong Mie scattering within the TiO_2_ microsphere, the optical path length increases thereby increasing the interaction with sensitizer. The scattering characteristics of photoanode were evaluated using diffuse reflectance spectroscopy (DRS). A comparison of diffuse reflectance spectra is shown in Fig. S2(b). The P25-TiO_2_ and SμS-TiO_2_ are compositionally and structurally similar as inferred from the estimated bandgap values (Fig. S2c) and XPS studies (Fig. S4). SμS-TiO_2_ shows higher light scattering than P25-TiO_2_, despite the similarity in the composition and optical property. The reasoning for increased light scattering can be understood from the PL studies as discussed in detail in the following section.

The PL spectra of P25-TiO_2_ and SμS-TiO_2_ acquired at room temperature using a 325 nm incident UV laser are shown in Fig. 5c. Both spectra show two broad peaks ~425 nm and 520 nm respectively. The first peak centered at ~425 nm in PL spectra is ascribed to self-trapped excitations and the peak centered at 520 nm is ascribed to defect related trap states^52,53^. The PL spectra of SμS-TiO_2_ exhibit multiple spikes on top of the broad peaks observed similar to that of P25-TiO_2_. These spikes observed in the PL spectra of SμS-TiO_2_ are called whispering gallery modes (WGM)^54^. These modes arise due to the total internal reflection of light at the oxide-air interface in microspheres. When a light of wavelength (λ) couples into the microsphere of size similar to the wavelength of incident light, resonance takes place giving rise to these WGM^55^. In order to investigate the effect of WGM on QD NBE emission, the PL spectra of CdSe QD loaded SμS-TiO_2_ were acquired using 488 nm laser line and the data obtained for different sized QD loaded on the photoanode are plotted in Fig. 5d. Interestingly these NBE spectra from QD also exhibit WGM spikes on top of the emission from the QD, implying a strong coupling between the WGM in SμS-TiO_2_ and the NBE emission of CdSe QD. These multi-peak sharp resonances from CdSe QD loaded SμS-TiO_2_ cover the whole visible spectrum which form the major component of Sun’s energy spectrum. Thus, WGM scattering from the SμS-TiO_2_ and the strong coupling with the CdSe QD can be used to enhance the light absorption in the sensitizer and increase the PCE of QDSWSC. Fabry-Perot cavity based resonant absorption has been demonstrated recently using colloidal QD. Ouellette *et al*.^56^ showed that by integrating the active layer of the photovoltaic device between two reflective interfaces the solar cells become sensitive for the spectral region at 1100–1350 nm, thus enabling effective IR light extraction. A schematic diagram of QDSWSC cartoon depicting the formation of WGM in SμS-TiO_2_ and its effect in PCE along with the FESEM image of SμS-TiO_2_ are given Fig. 5. The defect nature of CdSe QD loaded on the photoanode significantly influences the exciton formation and charge separation in solar cell. This influence was studied using photoconductivity measurements carried out in a van der Pauw four-probe configuration. The values of photoconductivity are in the order of 10^−8^ S/cm (Fig. S5a) and increase from 0.5 × 10^−8^ to ~5  × 10^−8^ S/cm with increasing QD size. The carrier generation lifetime and carrier recombination time are estimated and the values are an order of magnitude lower for the photoanode loaded with larger sized QD (Fig. S5b) further implying the deleterious role of defects in QD on the photovoltaic performance.

**Figure 6:**
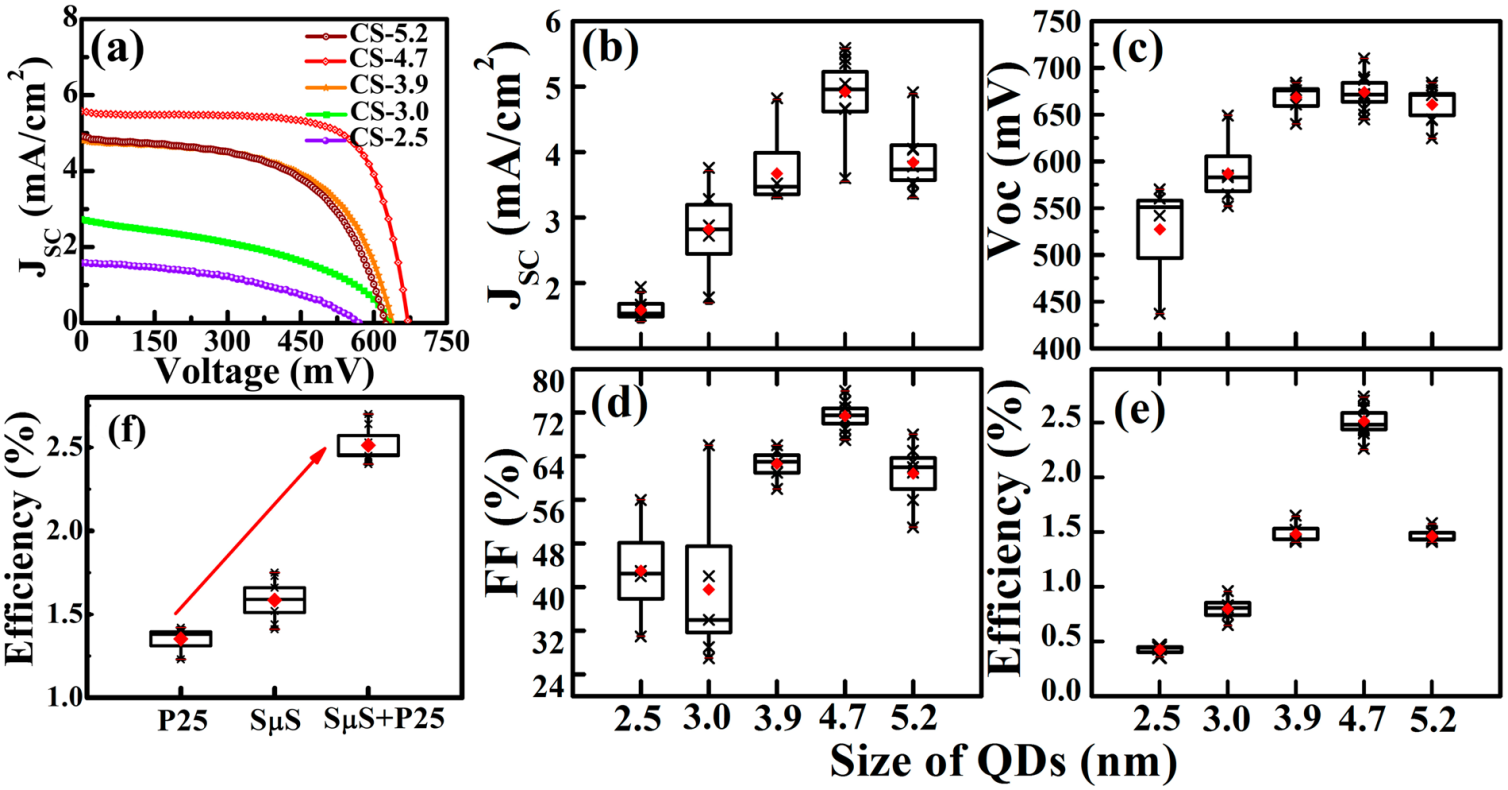
(**a**) J-V characteristic curve of QDSWSC devices based on different sized CdSe QD samples. Measurement was done under AM 1.5 G simulated full sunlight (100 mW/cm^2^) illumination. (**b**) to (**e**) are the characteristic parameters such as current density (J_SC_), open circuit voltage (V_OC_), fill factor (FF %) and efficiency (η%), respectively, of multiple number of QDSWSCs devices tested under similar condition. (**f**) represent the distribution plot for the efficiency of QDSSC made of P25-TiO_2_, and QDSWSC devices made of SμS-TiO_2_ and the composite (P25 + SμS)-TiO_2_ photoanodes using CS-4.7 QD. In the distribution plots (**b**) to (**f**), filled diamond in red color is the mean and the middle horizontal line in the box indicates the median of the data. The box range is selected as standard deviation and the vertical whisker line connected with maximum and minimum value of the data points. The cross symbols (⨉) on the whisker line are the data from number of devices tested using respective photoanode.

The TiO_2_ photoanodes made using microsphere-nanocrystallites composite is used to fabricate the QDSWSC. The thickness of fabricated TiO_2_ photoanode is ~20 μm and the cell area of device is 0.25 cm^2^. The CdSe QD are loaded on composite photoanode by electrophoretic method^32^ and then dipped in MPA solution for ligand exchange to enable proper link of CdSe QD with TiO_2_. The current density-voltage (*J-V*) characteristics of these CdSe sensitized QDSWSC measured with a 1 Sun illumination are shown in Fig. 6a. The photovoltaic characteristic parameters derived from these plots for several devices are summarized in distribution plots Fig. 6b–e. The J_SC_ of CdSe sensitized devices tend to increase with QD size except for the photoanode loaded with 5.2 nm sized CdSe QD. In all cases, V_OC_ exhibited values ranging from 570 mV to 684 mV and the FF values changes from 42 to 67. The J_SC_, FF and PCE of the devices show the increasing trend with size of CdSe QD. The highest PCE of ~2.74% is obtained for 4.7 nm CdSe QD loaded photoanode with photocurrent density (J_SC_) of ~5.6 mA/cm^2^. Enhanced efficiency is primarily due to the increased absorption by the CdSe QDs when sensitized on the composite photoanode (Fig. S6). This can also be seen from the IPCE measurements where the photon-to electron conversion efficiency has increased manyfold for the devices fabricated using composite photoanode (Fig. S7). Several QDSWSC devices were tested and the generic observation on the photovoltaic parameters is summarized in Fig. 6b–e. These devices also show low interfacial charge transfer resistance and recombination rate (Figs S8 and S9). The increase in PCE with CdSe QD size thus signifies less recombination due to decrease in surface defects thus enabling the delivery of higher photocurrent. It should be noted that, the PCE of QDSWSC devices are about 2 to 3 times more than the values reported for MPA capped CdSe, whereas these values are similar to S^2─^ capped CdSe QD^32^. Thus from these observation we establish that having a synergetic effect of high quality CdSe QD sensitized on a composite photoanode exhibiting WGM enhance the PCE significantly (Figs 6f and S10). Since the WGM multi-peak sharp resonances cover the whole visible region, the Mie scattering concept can be used to enhance the light absorption in the sensitizer, thus effectively enhancing the PCE of QDSWSC devices.

## Conclusion

Bandgap tailored cubic zinc-blende CdSe QD of various sizes were synthesized by hot-injection method. Surface disorders are shown to dominate in smaller sized CdSe QD. Photovoltaic performances of QDSWSC devices are studied by loading these CdSe QD on (SμS + P25)-TiO_2_ composite photoanode. The composite photoanode providing light scattering in the form of WGM (from SμS-TiO_2_) enable strong light absorption by the sensitizer. Nanocrystalline P25-TiO_2_ provide large surface area for sensitizer loading and better connectivity among microsphere. The increase in photovoltaic performance observed with QD size suggests the importance of minimizing the surface defects. We observe an efficiency of 2.74% for QD of size 4.7 nm, which is fivefold higher than the efficiency observed with devices sensitized with 2.5 nm QD (0.46%). WGM is shown to assist in increasing the efficiency by two fold. Thus, we establish by synergistically combining the approaches to control the size and surface defect in QD, and enhancing the light absorption through whispering gallery modes in mesoporous microsphere TiO_2_, the efficiency of quantum dot sensitized whisperonic solar cells can be significantly enhanced.

## Experimental

### Chemicals

Cadmium oxide (CdO), Se powder, Octadecane (ODE) (90%), oleic acid (OA), Trioctylphosphine (TOP) were purchased from Sigma-Aldrich for CdSe preparation. Degussa P25-TiO_2_ nanocrystallite was supplied by Evonik Aeroxide. All of the chemicals were used without any further purification.

### Synthesis of CdSe quantum dots

Different sizes of CdSe QD with zinc blende structure were prepared by hot-injection technique reported elsewhere^57–59^. In brief, a homogeneous clear cadmium precursor solution was prepared by heating 0.51 g of CdO, 6.19 ml oleic acid and 70 ml of ODE solution at 250 °C under N_2_ atmosphere. Se precursor solution was prepared separately by dissolving selenium powder (2 mmol) in 5 ml of TOP and 10 ml of ODA by continuous stirring at room temperature. The Se precursor solution was then injected in to the hot Cd precursor solution while the temperature was maintained at 200 °C throughout the reaction. As the reaction proceeded the QD grew in size and the change in size can be judged by the solution colour change during the reaction. Various sizes of QD were obtained by acquiring aliquots of reaction solution at regular intervals of reaction time. These QD are labeled as CS-*d*, where “CS” denotes CdSe QD and “*d”* denotes the size of QD in nm estimated from XRD. The QD were purified by centrifuge technique using excess acetone in QD solution. The centrifuge was done at 2500 rpm for 20–30 min. The supernatant was removed and sedimented QD were dispersed in toluene.

### Characterization

CdSe QD were characterized by various spectroscopic and microscopic techniques. The crystal structure and phase of CdSe QD were investigated using X-ray diffraction (XRD) recorded on a X’Pert-Pro, Panalytical powder diffractometer (Cu-Kα radiation) and Raman spectra acquired at room temperature with a 488 nm laser source using Horiba-Yvon (HR 800 UV) micro-Raman spectrometer. The shape and size distribution of CdSe QD were examined by transmission electron microscopy (HRTEM: FEI Tecnai G^2^ T20). The absorption spectra were recorded using a double-beam mode JASCO V-570 UV-Vis-NIR spectrophotometer by loading the solution in standard quartz cuvette. The defect level and band-edge emission of quantum dots are measured using photoluminescence spectra recorded using JASCO FP-6600 spectrophotometer at 400 nm excitation source. The quantum yields of the CdSe samples were determined with respect to a standard solution (~10^−4^ M) of Rhodamine 6 G in ethanol (96% purity). The bandgap of CdSe QD with various size distributions were studied by diffuse reflectance spectroscopy (DRS) using an integrating sphere configuration present in the PVE 300 quantum efficiency measurement system. X-ray photoelectron spectroscopy (XPS) of TiO_2_ photoanode was performed using SPECS instrument with Mg-Kα x-ray source and PHOIBOS 100MCD analyzer at residual gas pressure of below 10^−8^ Pa. Photoconductivity measurement of photoanode loaded with QD were measured using van der Pauw four-probe method on samples with area of 0.25 cm^2^ and thickness of ~20 μm.

### Solar cell fabrication

Fabrication of photoanode^31^ was carried out by taking TritonX-100 binder (Alfa Aesar) and photoanode (80 *wt*. % SμS and 20 *wt*. % P25) composite powder in a 1:1 weight ratio and preparing the TiO_2_ paste using ethanol as solvent by mixing them using a mortar and pestle^28^. Before coating TiO_2_ paste on FTO electrode, TiCl_4_ pre-treatment was done by dipping FTO glass substrate for 1 h in 30 mM TiCl_4_ solution at 80 °C. The paste was then coated on FTO electrode using doctor blade technique, and then annealed at 400 °C for 3 h. The thickness of fabricated photoanode film is ~ 20 μm and the area is ~0.25 cm^2^. QD were loaded on TiO_2_ photoanode by electrophoretic bath method^60^, using FTO/TiO_2_ substrate as a positive and a platinum wire (Pt) as a negative electrode. The distance between these two electrodes was maintained at 1 cm. An electrolyte solution of QD prepared by dispersing the CdSe QD in 40 ml of 1:1 toluene:acetonitrile mixture was used. A DC voltage of 100 V was applied between the electrodes for 5 min followed by rinsing of photoanode with toluene. This procedural cycle was repeated for 5 times. Finally, ligand exchange was performed by dipping the FTO/TiO_2_/CdSe substrate in MPA dissolved methanol solution (10 mg/mL) for 1 h. After ligand exchange, the electrodes were cleaned in methanol, dried in vacuum and taken for device testing. Pt coated FTO obtained by DC-magnetron sputtering of Pt target was used as counter electrode. An electrolyte solution containing iodide/tri-iodide redox couple (0.5 M of LiI, 0.05 M of I_2_ and 0.5 M 4-TBP in acetonitrile) was used.

The photovoltaic properties of QDSWSC devices were measured by fabricating a sandwich of two electrodes filled with the electrolyte. The area of cells tested was ~0.25 cm^2^. A solar simulator with AM 1.5 filter from Photo Emission Tech (Model # CT50AAA) fitted with a Xenon source (300 W) was used to illuminate the devices. A Keithley 2400 source meter was used to measure the J-V response under dark and light illumination. Open-circuit voltage-decay (OCVD) measurements were obtained with CHI electrochemical workstation (SP-50, Biologic). The electrochemical impedance spectroscopy (EIS) was performed using CH instrument (CHI600E series) under dark conditions. A bias voltage of 0.6 V was used and EIS spectra were collected in the frequency range of 0.1 to 10^5^ Hz. The spectra were fitted with a suitable equivalent circuit using Z-view program (Scribner Associates Incorporated).

## Electronic supplementary material

Supplementary Information
