# Supplementary material for: Whispering Gallery Mode Enabled Efficiency Enhancement: Defect and Size Controlled CdSe Quantum Dot Sensitized Whisperonic Solar Cells
Source: Sci Rep. 2018 Jun 26;8:9709. doi: 10.1038/s41598-018-27969-y (PMC6018832; doi:10.1038/s41598-018-27969-y)
Supplement: Supplementary file 1 — Supplementary Information [file 41598_2018_27969_MOESM1_ESM.pdf]

Supplementary information

Whispering Gallery Mode Enabled Efficiency Enhancement: Defect and Size Controlled CdSe Quantum Dot Sensitized Whisperonic Solar Cells

Tapan Kumar Das, P. Ilaiyaraja and C. Sudakar*

Multifunctional Materials Laboratory, Department of Physics, Indian Institute of Technology Madras, Chennai-600036, India

*corresponding author email - csudakar@iitm.ac.in

EXPERIMENTAL:

Synthesis of TiO_2 microsphere (SμS) by solvothermal method: About 200 mL of ethanol was taken in a beaker and adjusted to pH 3 using glacial acetic acid. 0.25 moles of titanium (IV) iso-propoxide was added into the beaker and stirred vigorously to obtain a homogeneous solution. This solution was transferred to an autoclave and heated at 200 ^oC for 2 h. After the solvothermal treatment, the residual solvent in the reaction mixture was slowly evaporated to obtain a white powder. The white powder was washed with de-ionized water until it reached pH 7. The powder was then calcined at 450 ^oC for 3 h to obtain crystalline anatase TiO_2 nanoparticles.

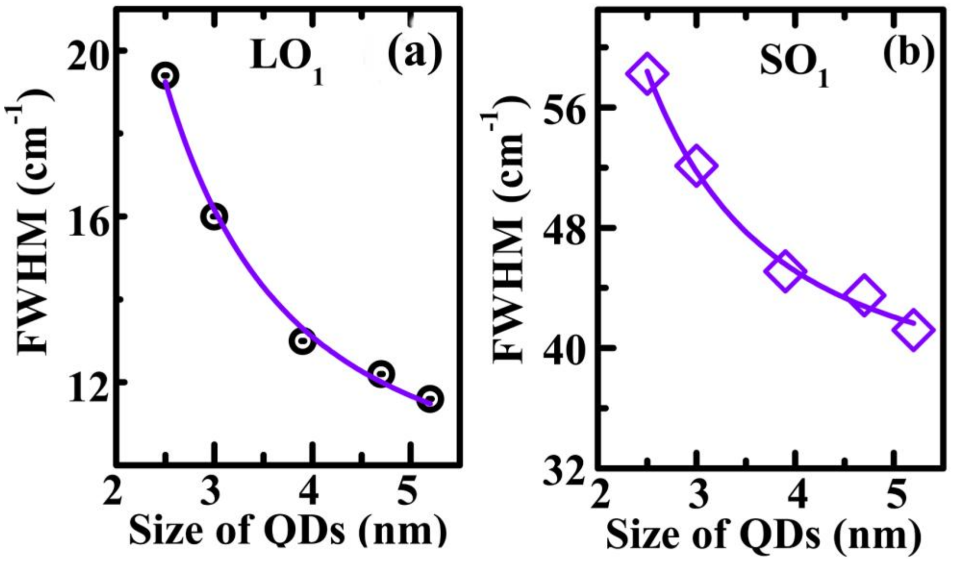

Figure S1. Size dependent variation of line width of (a) LO_1 and (b) SO_1 phonon modes.

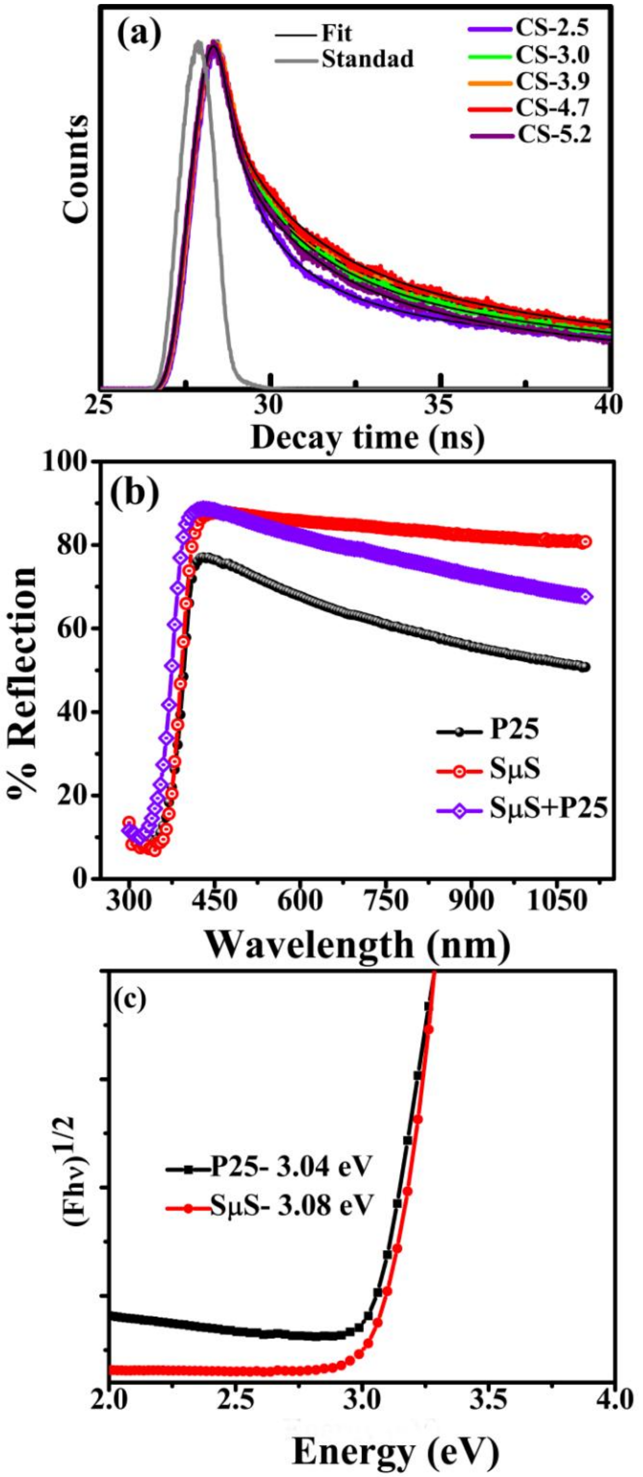

Figure S2. (a) PL lifetime decay of CdSe QD (b) Diffuse reflectance spectra of P25-TiO_2 and SμS-TiO_2 and their composite, (c) Tauc plots of P25-TiO_2 and SμS-TiO_2 nanostructure.

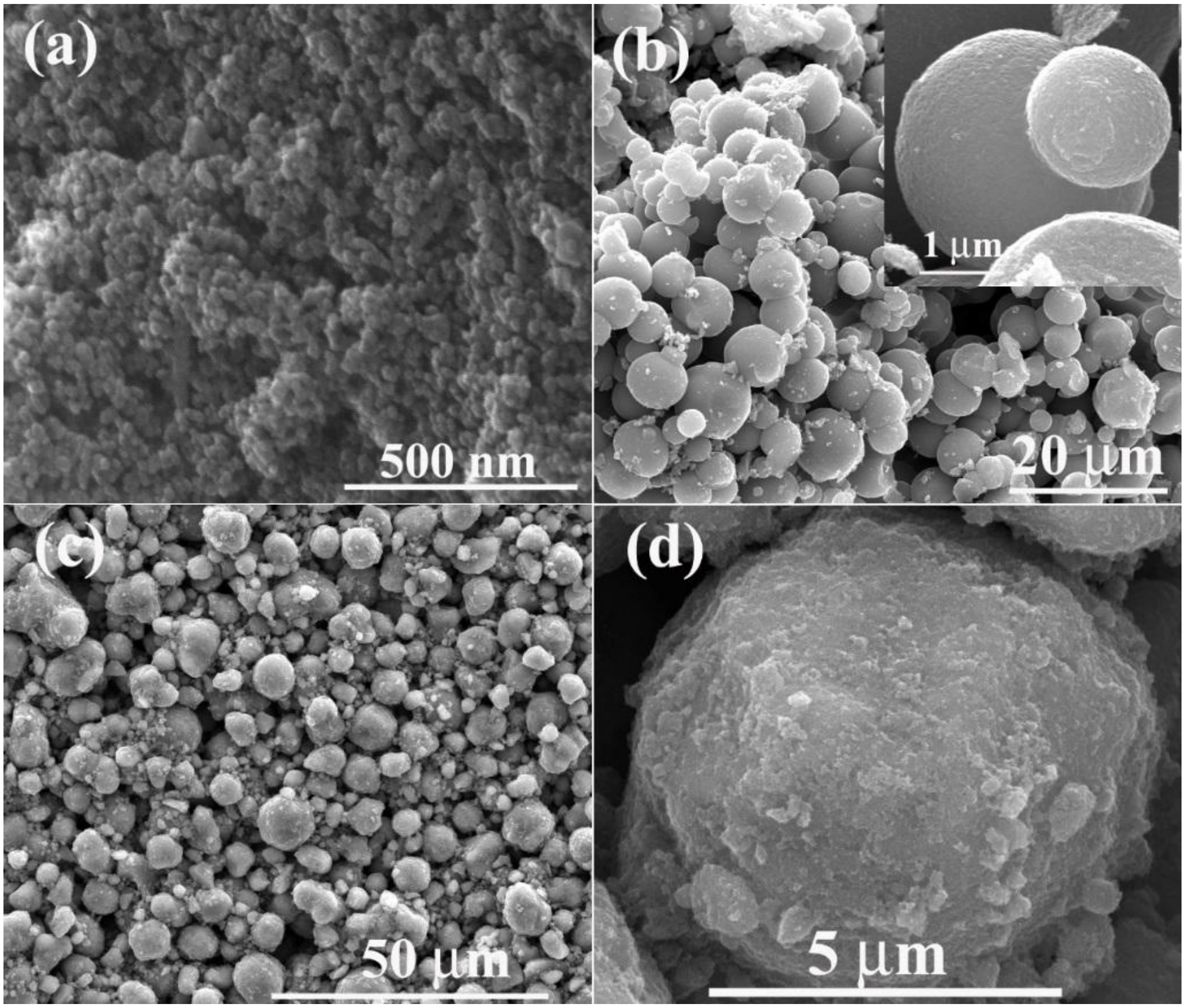

Figure S3. Field emission scanning electron microscope images of TiO_2 nanostructures; (a) P25-TiO_2 (Degussa); (b) smooth microspheres (SμS-TiO_2) and (c,d) the composite of nanoparticulate-microsphere TiO_2 (SμS+P25-TiO_2). The inset in (b) is a magnified image of SμS-TiO_2.

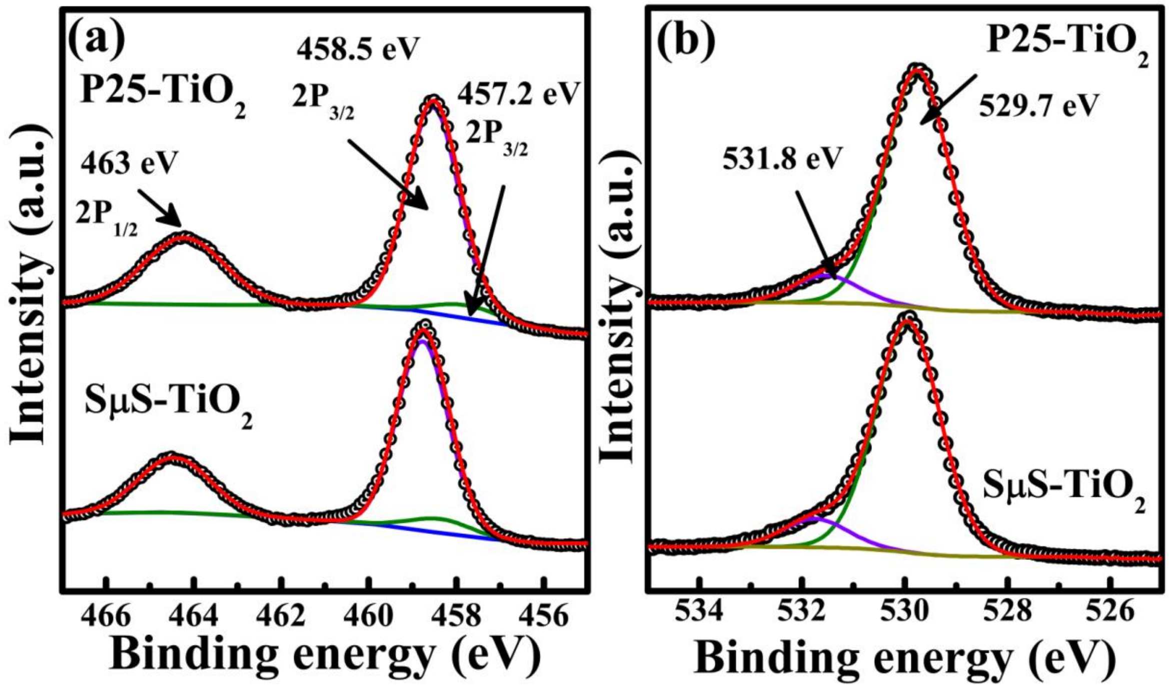

Figure S4. (a) Ti 2p and (b) O 1s XPS spectra obtained from P25-TiO_2 and SμS-TiO_2 photoanode; Deconvoluted peaks are shown in the green and blue color. The red line is the fit for the spectra.

XPS measurement carried out to understand the chemical states of both P25-TiO_2 and SμS-TiO_2 components are summarized in Fig.S4. The binding energies of Ti 2p and O 1s in TiO_2 lattice were calculated after correcting for the shift in the binding energy using C 1s edge as a reference. A doublet corresponding to the Ti 2p spin-orbit splitting (457.2 eV (Ti $2p_{3/2}$) and 463 eV (Ti $2p_{1/2}$)) is seen in the spectrum. The binding energy values of these peaks correspond to Ti^{4+} in TiO_2 lattice. However, the shoulder peak, resolved clearly when the profile fitting is carried out, positioned at 458.5 eV correspond to Ti $2p_{3/2}$ of Ti^{3+} species. This confirms a certain degree of nonstoichiometric nature of TiO_2 photoanode. The XPS study on P25-TiO_2 and SμS-TiO_2 indicate the presence of ~ 6 % of Ti^{3+} in TiO_2 (see Fig. S4(a)), which is mostly from surface region. The oxygen vacancies are also inferred from the secondary peaks of O 1s XPS

spectral lines. O 1s spectra are fitted into two peaks, which include crystal lattice oxygen (Ti-O, 529.7 eV) and the oxygen defect regions with lower coordination (531.8 eV) (see Fig. S4(b)). Certainly, the presences of Ti^{3+} in both TiO_2 are low, which is good for high performance photoanode in photovoltaic cell.

To see the effect of CdSe loading on the photoanode, which will significantly influence the exciton formation and charge separation in solar cell, photoconductivity measurements of composite photoanode loaded with various sizes of CdSe QD were carried out using van der Pauw four probe method. The time dependent change in the conductivity under the light ON and OFF (AM 1.5 one Sun illumination (100 mW/cm^2)) conditions are measured by monitoring the voltage change as a function of time at constant applied current using Keithley 2400 source meter. The conductivity of the photoanode increases on exposure to the light and gets saturated after ~10 seconds (Fig. S5a). When light is turned OFF, the conductivity decreases and get back to the original value. The recovery response is little slower than the response during the light ON condition indicating possible role of trapped states. The photoconductivity of photoanode loaded with different size of QD show significant differences in response. The values of photoconductivity for all the samples are in the order of 10^{-8} S/cm. This response is less in the case of photoanode loaded with smaller sized QD. The photoconductivity change ($\Delta\sigma_t$) for photoanode loaded with larger sized QD increases significantly. Using the equation $\frac{\sigma_t-\sigma_{\text{off}}}{\sigma_{on}-\sigma_{\text{off}}} = \frac{\Delta\sigma_t}{\Delta\sigma_{total}} = a(1-e^{-\frac{t}{\tau_g}})$, the average carrier generation lifetime (τ_g) from the rising part of the conductivity in photoanodes are estimated,[62] where $\Delta\sigma_t$ $(=\sigma_t-\sigma_{\text{off}})$ is the difference in the conductivity between the ON (σ_{on}) and OFF (σ_{off}) states and a is a constant. For the OFF state the change in conductivity is fitted with a stretched exponential function given by $\frac{\sigma_t-\sigma_{\text{off}}}{\sigma_{on}-\sigma_{\text{off}}} =$

$\frac{\Delta\sigma_t}{\Delta\sigma_{total}} = ae^{-\left[\frac{t}{\tau_r}\right]^b}$, where τ_r is the carrier recombination lifetime, a is a constant and b indicates the deviation from exponential function.[62] The carrier generation lifetime τ_g and carrier recombination τ_r are estimated as representatively shown in Fig. S5b for 2.5 nm sized QD loaded on TiO_2 photoanode. The values are found to be $\tau_g \sim 12.7\,s$ and $\tau_r \sim 10.6\,s$ for the photoanode loaded with QD of size 2.5 nm (see Table S1). These values decrease by an order for the photoanode loaded with larger sized QD. Thus, the presence of defects states in smaller sized QD are having strong effect on the recovery response time. Despite of having large number of defect states in smaller size QD, the carrier recombination lifetime is larger. When the light is turned OFF, the conductivity turns to its initial values, which is not spontaneous and takes longer time for smaller sized QD.

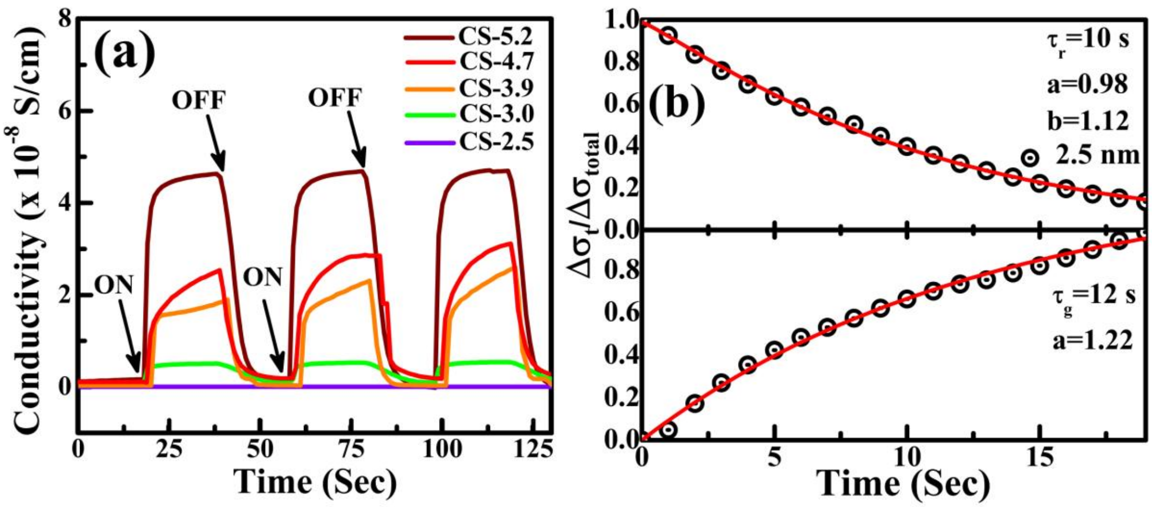

Figure S5. (a) Photoconductivity response of TiO_2 photoanode loaded with different size CdSe QD under the ON and OFF states of AM 1.5 source and (b) show the increasing and decreasing segments of photoconductivity response, the open symbols show the measured data points and solid line is the exponential fit.

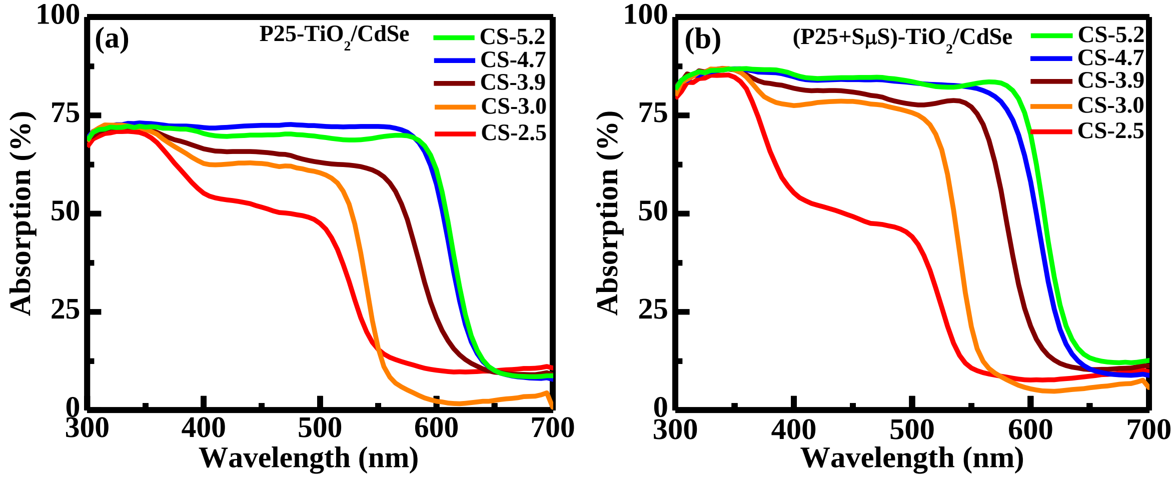

Figure S6. Absorption spectra of CdSe QDs loaded (a) on P25-TiO_2 and (b) on P25+SμS-TiO_2 composite. Absorption spectra were collected by keeping the QD loading same for all photoanode.

The absorption by the CdSe QDs increases after sensitizing the TiO_2 with CdSe QD. This enhancement in the absorption (%) is found be to more for (P25+SμS)-TiO_2 than P25-TiO_2 photoanode (Fig. S6). This also directly correlates with the increase in the efficiency observed as reported in the manuscript. The spectra were obtained by keeping the thickness of photoanode and loading of CdSe QDs similar for all the samples. The absorption spectra of CdSe QDs loaded on (a) P25-TiO_2 and (b) (P25+SμS)-TiO_2 composite show systematic increase in absorption characteristics. With increase in CdSe QDs size, the absorption increases in both the photoanode, however, the increase is much higher for the (P25+SμS)-TiO_2 composite photoanode. It should be noted that the absorption maxima and characteristics absorption of CdSe QDs remain same for both the photoanode, however the absorption (%) is distinctly found to be more for CdSe QDs loaded on (P25+SμS)-TiO_2 photoanode. Thus, the enhancement in overall absorption in CdSe QDs loaded on composite photoanode lead to the enhanced

efficiency. The convincing evidence for the increased absorption due to the whispering gallery modes can be directly evidenced from the emission characteristics of QDs loaded on P25-TiO_2 and the SμS-TiO_2 as detailed in main manuscript.

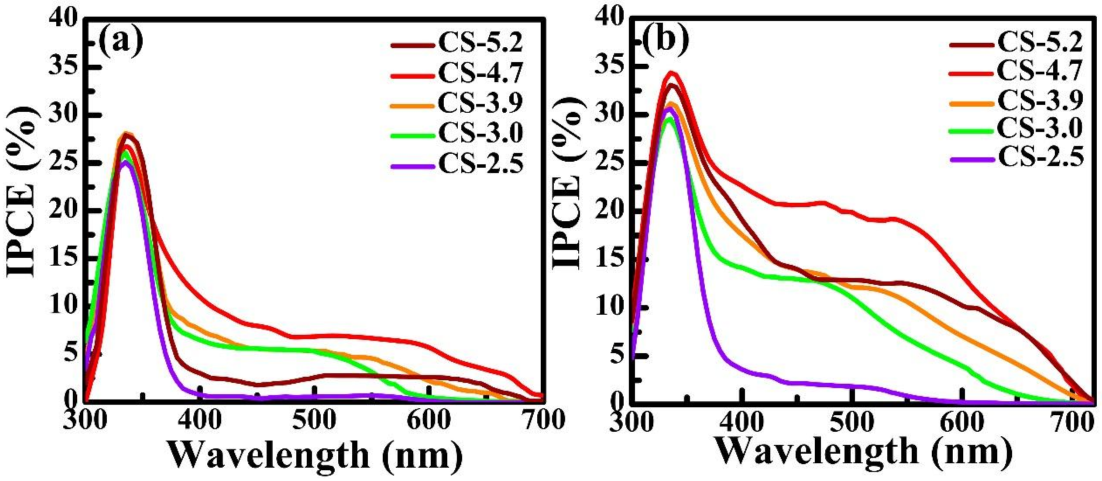

Figure S7. IPCE spectra of CdSe QDs loaded (a) on P25-TiO_2 based photoanode QDSSC and (b) on (P25+SμS)-TiO_2 composite photoanode QDSWSC. The spectra were collected by keeping the QD loading same for all devices.

The incident photon-to-current conversion efficiency (IPCE) was measured using Bentham PVE300-Quantum efficiency measurement system without bias light. IPCE of QDSSC devices made from P25-TiO_2 photoanode and the QDSWSC devices made using (P25+SμS)-TiO_2 composite photoanode are presented in Fig. S7.

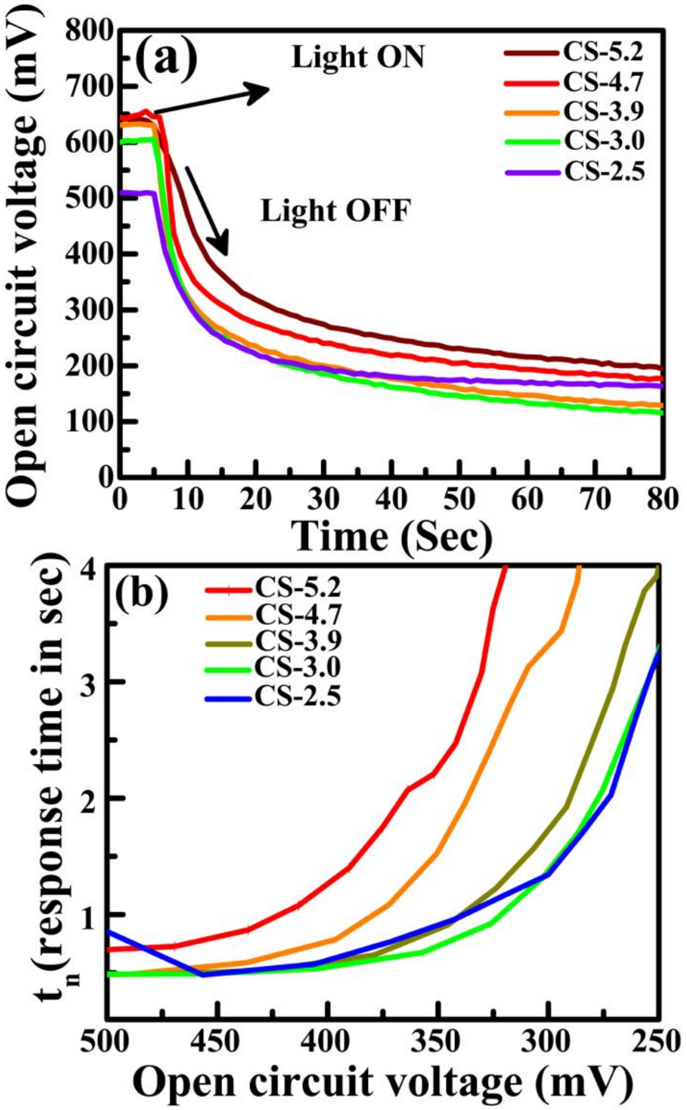

Figure S8. (a) Open-circuit voltage decay measurement of the QDSWSC based on (P25+SμS)-TiO_2 composite photoanode with different sized CdSe QD, (b) The electron response time derived from the equation (1) as a function of V_{OC}.

The recombination kinetics of the QDSWSC devices are investigated from the open-circuit voltage-decay (OCVD) measurements (Fig. S8). The OCVD measurement of the QDSWSC with different size of CdSe QD was carried out by initially exposing the device with AM1.5, one Sun illumination (100 mW/cm^2) condition for 10 seconds and then with the light turned OFF conditions V_{OC} is recorded as a function of time. The OCVD plots are shown in Fig.

S8a. The OCVD curves can be categorized in to three different regions.[58] The high voltage region correspond to free electron, the middle exponential decay region is related to the internal trapping of charge carriers in the photoanode material and the low photovoltage parabolic region correspond to the density of the acceptor in electrolyte. The main region of interest is the middle exponential decay, which relates to the electron recombination process in the anode and electron lifetime.[59] The response time of electron after switching OFF the light is estimated using the transient equation given below (also see Fig. S8b)[59]

$$\tau_n = \frac{-K_B T}{e}\left[\frac{dV_{OC}}{dt}\right]^{-1} \quad (1)$$

where K_B is the Boltzmann constant, T is the temperature, e is the elementary charge of electron and $\frac{dV_{OC}}{dt}$ is the derivative of the open circuit voltage transient. The response time of electron in the device made of photoanode sensitized with smaller size CdSe QD is corresponding to the middle regime is smaller than the device with larger sized QD. Longer response time of electron observed in this regimes in larger QD sensitized device could be due to the lower concentration recombination center and it is noteworthy that such long response time provide better pathway for electron transport.

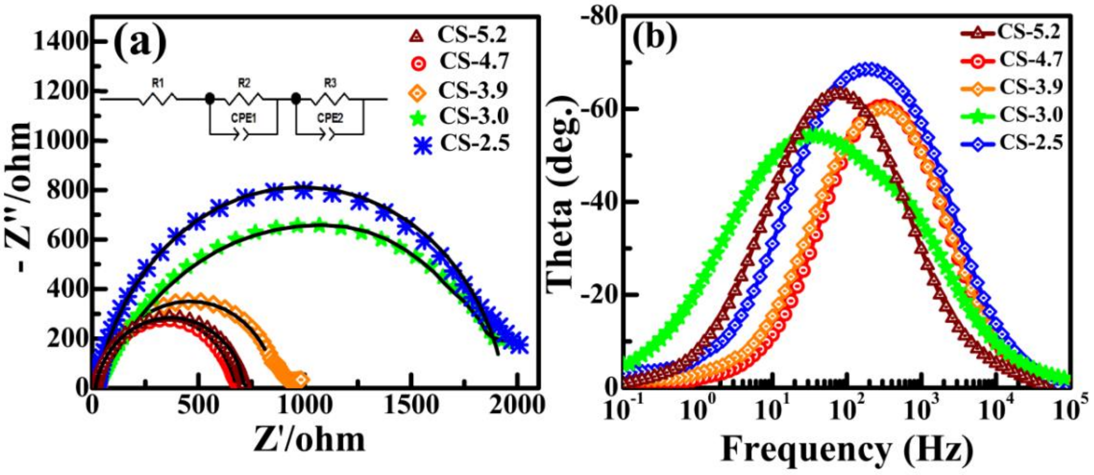

Figure S9. EIS spectra of QDSSC device based on different sized CdSe QD samples. (a) The Nyquist plots fitted with an equivalent circuit given as inset are shown by the overlapping lines plotted on the data points and (b) is the corresponding Bode plots obtained from EIS study.

The charge transfer resistance at the interface also dictates the overall cell performance of the device. The charge transfer resistance at the interface is estimated from electrochemical impedance spectroscopy (EIS) studies. The measurements were carried out in the dark under 0.6 V negative bias and a frequency range from 0.1 Hz to 10^5 Hz. The results are presented in the Nyquist and Bode plots shown in Fig. S9a and Fig. S9b, respectively. The spectra are fitted with simulated curves corresponding to an equivalent circuit made of two parallel RC components obtained using Z-view software.[64] The simulated EIS spectra agree well with the experimental curves. Each RC element contains resistance (R) and constant phase elements (CPE). The resistance R_1 is attributed to contact resistance between FTO/Pt and is found to be 20-40 Ω for all the devices. The sensitizer-oxide/electrolyte interface resistances R_2 differ depending on the size of QD sensitized on the photoanode. This resistance is found to be an order more in the case of 3.0 nm and 2.5 nm QD sensitized photoanode implying more surface defects present in the QD make the interface resistive to the charge flow, which result in the smaller current density

(J_{SC}) in the device. The interfacial capacitance C_{int} due to charge transfer and recombination at interface sensitizer-oxide/electrolyte are estimated. The interfacial capacitance, C_{int}, due to charge transfer and recombination at the sensitizer/oxide/electrolyte interface are found to be less in CS-3.9, CS-4.7 and CS-5.2 samples. This indicates that the electron lifetime ($\tau_n = R_2 C_{int}$) is found to be less in larger sized QD. All the fitted parameters are given in the Table 3. The resistances R_3 at the electrolyte/counter electrode (Pt/FTO) interface show very minimal change in all the devices and are similar to the contact resistance between FTO/Pt. No striking difference in the impedance spectra Bode phase plots (Fig. S9b) are discerned and all the spectra show two characteristic frequency responses with slight increase in characteristic frequency in CS-3.9 and CS-4.7 samples, suggesting a shortened electron lifetime.

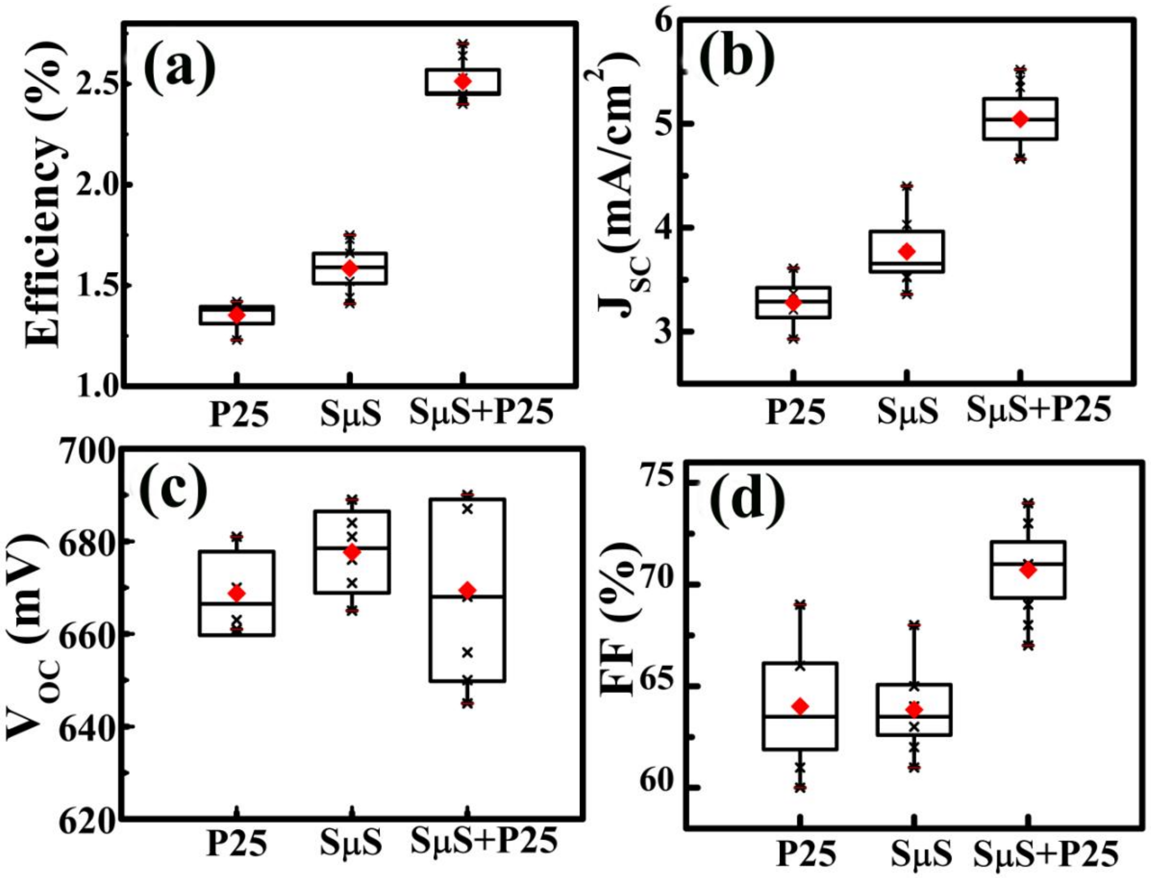

Figure S10. The photovoltaic parameters [(a) Efficiency, η (b) current density, J_{SC}, (c) open circuit voltage, V_{OC}, and (d) fill factor, FF] of QDSSC made of P25-TiO_2 photoanode, and the QDSWSC device made of SμS-TiO_2 and their composite (P25+SμS)-TiO_2. The devices are made by sensitizing the photoanode with CS-4.7 QDs. The filled diamond in red color is the mean and the middle horizontal line in the box indicates the median of the data. The box range is selected as standard deviation and the vertical whisker line connects the maximum and minimum value of the data points.

Table S1: The characteristics parameters of QDSSCs such as current density (J_{SC}), open circuit voltage (V_{OC}), fill factor (FF) and efficiency (η) made using different size of CdSe QDs and the estimated interfacial resistance of the device from electrochemical impedance spectra are also given in the table.

Sample code	J_{SC} (mA/cm^2)	V_{OC} (mV)	FF (%)	η (%)	Conductivity (× 10^{-8} S/cm)	Conductivity		Estimated resistances of EIS circuit elements			
						τ_g (Sec)	τ_r (Sec)	R_1 (Ω)	R_2 (Ω)	R_3 (Ω)	C_{int} (×10^{-5} F)
CS-5.2	3.16	624	56	1.67	4.6	2	5.5	15	699	1	0.20
CS-4.7	5.6	672	71	2.74	2.5	2.8	5.3	24	640	16	0.48
CS-3.9	4.8	640	58	1.75	1.9	2.1	5.9	39	720	80	0.61
CS-3.0	2.7	649	55	0.96	0.5	7.2	5.1	23	1966	14	3.42
CS-2.5	1.6	570	50	0.46	0.05	12.7	10.6	22	1973	13	3.37
